# Two to five repeated measurements per patient reduced the required sample size considerably in a randomized clinical trial for patients with inflammatory rheumatic diseases

**DOI:** 10.1186/1756-0500-6-37

**Published:** 2013-02-01

**Authors:** Geir Smedslund, Heidi Andersen Zangi, Petter Mowinckel, Kåre Birger Hagen

**Affiliations:** 1 National Resource Centre for Rehabilitation in Rheumatology, Diakonhjemmet Hospital, P.O. Box 23 Vindern, Oslo, 0319, Norway

**Keywords:** Sample size, Statistical power, Clinical trial, Arthritis, General health questionnaire

## Abstract

**Background:**

Patient reported outcomes are accepted as important outcome measures in rheumatology. The fluctuating symptoms in patients with rheumatic diseases have serious implications for sample size in clinical trials. We estimated the effects of measuring the outcome 1-5 times on the sample size required in a two-armed trial.

**Findings:**

In a randomized controlled trial that evaluated the effects of a mindfulness-based group intervention for patients with inflammatory arthritis (n=71), the outcome variables Numerical Rating Scales (NRS) (pain, fatigue, disease activity, self-care ability, and emotional wellbeing) and General Health Questionnaire (GHQ-20) were measured five times before and after the intervention. For each variable we calculated the necessary sample sizes for obtaining 80% power (α=.05) for one up to five measurements.

Two, three, and four measures reduced the required sample sizes by 15%, 21%, and 24%, respectively. With three (and five) measures, the required sample size per group was reduced from 56 to 39 (32) for the GHQ-20, from 71 to 60 (55) for pain, 96 to 71 (73) for fatigue, 57 to 51 (48) for disease activity, 59 to 44 (45) for self-care, and 47 to 37 (33) for emotional wellbeing.

**Conclusions:**

Measuring the outcomes five times rather than once reduced the necessary sample size by an average of 27%. When planning a study, researchers should carefully compare the advantages and disadvantages of increasing sample size versus employing three to five repeated measurements in order to obtain the required statistical power.

## Background

Patient reported outcomes (PRO) are accepted as important outcome measures in rheumatology. In patients with rheumatic diseases the symptoms are fluctuating [1]. This has serious implications for sample sizes in clinical trials. Since the within-patient variation will be included in the total variation, this in turn will increase the standard deviation and finally require larger sample sizes.

When planning a clinical trial, the researchers want to design it so that it has sufficient statistical power to detect a clinically relevant difference between groups [2]. When researchers draw conclusions, they want to avoid committing a Type II error, which basically means concluding that there is no difference when there is in fact a difference. Power is the probability of not making a Type II error. The Type II error probability (β) is by convention set to .2, which equals a power of 80 percent. Most often researchers will compute the sample size necessary for detecting the minimal clinically important difference (MCID) when planning a trial. At the same time, they need to minimize the Type I error probability to avoid concluding with an effect when the null hypothesis is true. The required sample sizes may often be hard to obtain for different reasons. Keen et al. investigated the prevalence of underpowered RCTs in rheumatology, and found that a substantial number of randomized controlled trials of adult rheumatic diseases were underpowered [3]. Approximately 50% of the RCTs with negative results were underpowered, and these trials may not have been of sufficient sample size to detect clinically meaningful treatment effects.

One way of increasing power is to reduce the standard deviation (SD) of the outcome of interest. This can be achieved by measuring the outcomes on several occasions on the same patient and averaging the measurements. Thus, it is possible to increase power without including additional patients in the trial. In a previous prospective observational study we simulated how the within-subject variation decreases when the number of measurements is varied between 1 and 42 [2]. Considering the findings that five measurements per patient can be optimal for reducing the SD [2], we decided to employ this concept in a recently published trial [4]. The aim of the present study was to show how much the required sample size decreased when the number of measurements was increased from one up to five.

## Methods

In a randomized controlled trial that evaluated the effects of a mindfulness-based group intervention for patients with inflammatory arthritis (n = 71), the primary outcome variable was psychological distress measured by the General Health Questionnaire (GHQ-20), whereas secondary outcomes included pain, fatigue, disease activity, self-care ability, and emotional wellbeing measured by Numerical Rating Scales (NRS) [4]. The items on the GHQ are scored on a four-point Likert scale (0 to 3) which gives a possible sum score of between 0 (no distress at all) and 60 (much more distress than usual) [5,6] The GHQ-20 has been validated and used in various samples of chronically ill persons in Norway. [4]. The NRS is a 10 cm horizontal line, numbered from zero to ten and anchored with two extremes at the ends (e.g. 0 = no pain/fatigue and 10 = intolerable pain/fatigue). The respondents are asked to tick the number that best indicate their condition [7]. During the baseline period each variable was measured at two-week intervals five times. The first four measurements were conducted by telephone interviews, and the fifth measurement by a questionnaire sent to participants with a postage-paid return envelope.

Although there are several possible statistical methods available for repeated measurements, we chose to do it as simple as possible by computing a pooled mean. Thus, for each outcome measure, we calculated the SD attained by pooling two, three, four, and five measurements as compared to the single measurement. We calculated required sample sizes assuming α=.05 and β=.2 (power: 80%). Because there were no clear recommendations in the literature about what a clinically relevant change in the GHQ-20 might be, we performed a pilot study prior to the RCT. Based on the results of this pilot, we hypothesised that the RCT would detect a difference between groups of 4.5 in GHQ-20 with an estimated SD of 3.9, and a probability of a slight improvement of 0.9 in the control group. Thus, in the present study the value of 4.5 was used as a minimal clinical important difference (MCID) for the GHQ-20, and a MCID of 1 was used for the NRS scales. For each variable we calculated the necessary sample sizes for obtaining 80% power both for the single measurement case and for the two- three-, four-, and five measurement cases. In order to check whether choice of MCID would alter the results, we ran additional analyses using MCIDs of 6 for the GHQ and 2 for the NRS scales.

## Findings

**Figure 1:**
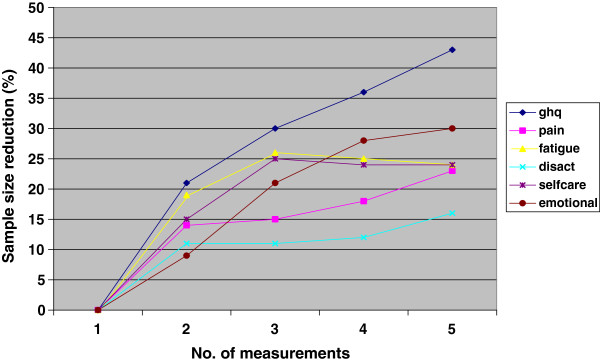
Percent reduction in required sample size as a function of increasing number of measurements per patient.

Measuring the outcomes five times rather than once reduced the necessary sample size by an average of 27% for all outcomes (Figure 1). Using two, three, and four measurements reduced the required sample size by 15%, 21%, and 24%, respectively. When we measured the outcomes three (five) times, the sample size per group was reduced from 51 to 39 (32) for the GHQ-20, from 74 to 60 (55) for pain, 99 to 71 (73) for fatigue, 68 to 51 (48) for disease activity, 66 to 44 (45) for self-care, and 52 to 37 (33) for emotional wellbeing (Table 1). Using different MCIDs produced essentially the same results (data not shown).

**Table 1:** Standard deviation and required sample size (percent reduction) as a function of 1–5 measurements

**Standard deviation**					
**Variable**	**1 measure**	**2 measures**	**3 measures**	**4 measures**	**5 measures**
GHQ	8.44	7.51	7.01	6.76	6.39
Pain	2.11	1.97	1.95	1.92	1.86
Fatigue	2.47	2.22	2.12	2.13	2.15
Disease activity	1.89	1.80	1.79	1.77	1.73
Self-care	1.92	1.77	1.67	1.69	1.69
Emotional well-being	1.73	1.65	1.52	1.47	1.43
**Required sample size (percent reduction) per group**					
**Variable**	**1 measure**	**2 measures**	**3 measures**	**4 measures**	**5 measures**
GHQ	56	44 (21)	39 (30)	36 (36)	32 (43)
Pain	71	61 (14)	60 (15)	58 (18)	55 (23)
Fatigue	96	78 (19)	71 (26)	72 (25)	73 (24)
Disease activity	57	51 (11)	51 (11)	50 (12)	48 (16)
Self-care	59	50 (15)	44 (25)	45 (24)	45 (24)
Emotional well-being	47	43 (9)	37 (21)	34 (28)	33 (30)

## Discussion

To our knowledge this is the first study within rheumatology using empirical data from a randomized controlled trial to estimate the effects of 1–5 measurements on the required sample size. We found that five measurements reduced the required sample size by 27% on average. Considering that a substantial number of randomized controlled trials of adult rheumatic diseases were underpowered [3], researchers should carefully consider the advantages of employing five measurements in order to obtain the required statistical power. However, a major reduction (21%) was achieved by three measurements only. Introducing a fourth and fifth measurement reduced the required sample size relatively less. In fact, for two outcomes (fatigue and self-care) the standard deviation and the required sample size increased marginally when adding a fourth and fifth measurement. For all other outcomes there was a monotonic decreasing standard deviation and required sample size as the number of measurements was increased up to five. Thus, empirical data from the current trial indicate that three measurements per patient can be beneficial from a cost perspective.

A possible explanation for the two exceptions to our general findings described above can be found in an article by Winkens et al. [8]. They reported that the covariance structure can greatly influence the optimal number of measurements. In their data, two measurements often yielded highly efficient treatment effect estimators. More measurements were needed if a) the covariance structure was compound symmetric or b) the structure approached compound symmetry (CS) and the correlation between two measurements did not exceed 0.80 or c) the correlation did not exceed 0.60, if the time lag went to zero. Zhang and Ahn [9] also found that more measurements are needed under the CS correlation and that the magnitude of sample size reduction quickly decreases when the number of measurements per subjects increases beyond 4.

It seems that five measurements have a larger effect on the required sample size for the GHQ than for the NRS scales. This may be because the GHQ consists of 20 items, each of which have a variance, while the NRS is a single-item scale.

Some limitations of using repeated measurements need to be considered. Completing questionnaires may be both time consuming and strenuous for the patients. The benefit of increasing the number of measurements should therefore be balanced against the burden on the respondents. In the present study, we decided to use telephone interviews for four of the measurements, assuming that the burden on patients would be less if they were called than if they were asked to fill in and return questionnaires, and that this would increase the response rate. The interviews may have introduced a recall bias and thereby influenced the within-person variation.

Another limitation is the costs of completing the measurements in terms of time and personnel needed, which should be balanced against the costs of including a larger sample. Also the choice of MCID can be discussed. However, the size of MCID did not influence the relative reduction in sample size in our analyses.

We end with a cautionary note. As one of the reviewers pointed out: “…when planning a longitudinal study with repeated measurements, researchers must estimate sample size a priori (by fixing alpha, beta, MCID, number of repeated measurements, variability of the outcome, correlation (correlation structure)”. In other words, they have to assume a model of the data. This means that other researchers cannot simply apply our reported empirical sample size reductions for other outcomes in other contexts.

## Conclusions

We found that using up to three measurements per patient substantially reduced the required sample size for all measures in this study. Adding a fourth or fifth measure resulted in a smaller reduction. When planning a trial, researchers should carefully compare the advantages and disadvantages of increasing sample size versus employing multiple measurements in order to obtain the required statistical power.

## Competing interests

All authors declare they have no competing interests.

## Authors’ contributions

All authors contributed to the planning of this study. HZ and KBH collected the data, and GS and PM analysed the data. GS wrote the first draft of the article. All authors read and provided feed-back on the draft-versions of the article, and approved the final version. GS is the guarantor.

## Source of funding

This work was funded by the National Resource Centre for Rehabilitation in Rheumatology.
